# Femoral vein pulsatility: a simple tool for venous congestion assessment

**DOI:** 10.1186/s13089-023-00321-w

**Published:** 2023-05-10

**Authors:** V. Bhardwaj, P. Rola, A. Denault, G. Vikneswaran, R. Spiegel

**Affiliations:** 1 Critical Care, Narayana Health City, Bangalore, India; 2 ICU Chief of Service, Santa Cabrini Hospital, Montreal, Canada; 3 Department of Anesthesiology, Monteal Heart Institute, Montreal, Canada; 4 Clinical Research, Narayana Health City, Bangalore, India; 5 Department of Critical Care Medicine, Medstar Washington Hospital Center, Washington, DC USA

**Keywords:** Venous congestion, VExUS, Central venous pressure, Fluid management, Monitoring

## Abstract

**Background:**

Femoral vein Doppler (FVD) is simpler than the VExUS score which is a multimodal scoring system based on combination of IVC diameter, hepatic venous Doppler, portal vein pulsatility and renal vein Doppler, may be useful in assessing right ventricular overload and signs of venous congestion. There is limited data on the relationship between FVD and VExUS score.

**Results:**

Adult post-cardiac surgery patients were assessed for venous congestion using the VExUS score and FVD. Agreement between VExUS and FVD was studied using Kappa test, sensitivity, specificity, PPV and NPV for VExUS and FVD was calculated keeping CVP as gold standard. In total, 107 patients were enrolled, with a mean age of 55.67 ± 12.76. The accuracy of VExUS and FVD for detecting venous congestion was 80.37 (95% CI of 71.5 to 87.4) and 74.7 (95% CI of 65.4 to 82.6), respectively. The level of agreement between FVD and VExUS was moderate (Kappa value of 0.62, *P < *0.001) while the agreement between FVD and CVP was weak (Kappa value of 0.49, *P < *0.001).

**Conclusion:**

FVD has good accuracy for detecting venous congestion and shows moderate agreement with VExUS grading. With potentially easier physical accessibility and a shorter learning curve for novices, it may be a simple and valuable tool for assessing venous congestion.

**Supplementary Information:**

The online version contains supplementary material available at 10.1186/s13089-023-00321-w.

## Introduction

In recent years, several groups have begun to shift focus onto the deleterious effects of venous congestion. Traditional assessment of right heart congestion such as peripheral edema, input and output charting and weight have limited accuracy and may not reflect intravascular venous hypertension. While jugular venous distension may be more applicable, it can be difficult to reliably assess, and while central venous pressures (CVP) has been well correlated to poorer outcomes [1], it requires invasive monitoring. Point-of-care ultrasound is an excellent non-invasive tool to assess physiology at the bedside, and lends itself very well to vascular flow assessment. The group of Beaubien-Souligny et al. created a composite score, the venous excess ultrasound or VExUS score, which correlated with increased levels of renal dysfunction in post-op cardiac surgery patients, and subsequent observational studies corroborated this association [2–4], with interventional studies currently underway. However, Denault et al. suggested that femoral vein Doppler (FVD), which is simpler than the VExUS score, may also be useful in assessing right ventricular overload and signs of venous congestion [5]. The relationship between FVD and VExUS score has not been reported. In this study, we compare FVD to the VExUS score in order to establish their correlation.

## Methods

This is a prospective observational study conducted in the adult post-cardiac surgical unit of a tertiary care center. The center is one of the largest cardiac centers in India which specializes in minimally invasive coronary bypass, complex valve surgeries, pulmonary thromboendarterectomy and cardiac transplantation. All adult consecutive post-cardiac surgery patients were included for the study. Patients with inadequate window for USG, in respiratory distress (respiratory rate > 35/min, accessory muscles of respiration in use), liver cirrhosis, deep vein thrombosis of lower limb, pregnant women were excluded from the study. The study was approved by the ethics committee (NH/AEC-CL-2022-833) and waiver of consent was obtained. Baseline demographic details were obtained from medical records. USG examination for VExUS score, femoral vein pulsatility were done by an trained and intensivist with more than 5 years’ experience in bedside ultrasound. All images were reviewed and scores were confirmed by another intensivist with extensive bedside ultrasound experience. Any discrepancy in score were discussed and resolved.

Ultrasound assessment was performed bedside using a Sonosite M Turbo machine using a cardiac probe (1–5 MHz) Patients were positioned in the dorsal decubitus position with the head of bed elevated at 30°. In the same position, CVP was recorded using a central venous catheter with the transducer positioned at mid thoracic level. Hepatic venous Doppler was done, visualizing either the middle hepatic vein in the subxiphoid area or the right hepatic vein from a lateral angle. Similarly, the portal vein Doppler was interrogated from a lateral approach. All Doppler examinations were recorded and measured at end-expiration during respiratory pause.

The VExUS score was obtained using the published guidelines detailed in Additional file 1.

All the patients had CVP line inserted through right internal jugular vein, none were in the femoral vein. All patients were monitored with CVP in which the transducer was zeroed at the mid-axillary point. Mean CVP value read on display was recorded. The mean CVP values were noted simultaneously to the femoral venous Doppler examination.

**Fig. 1:**
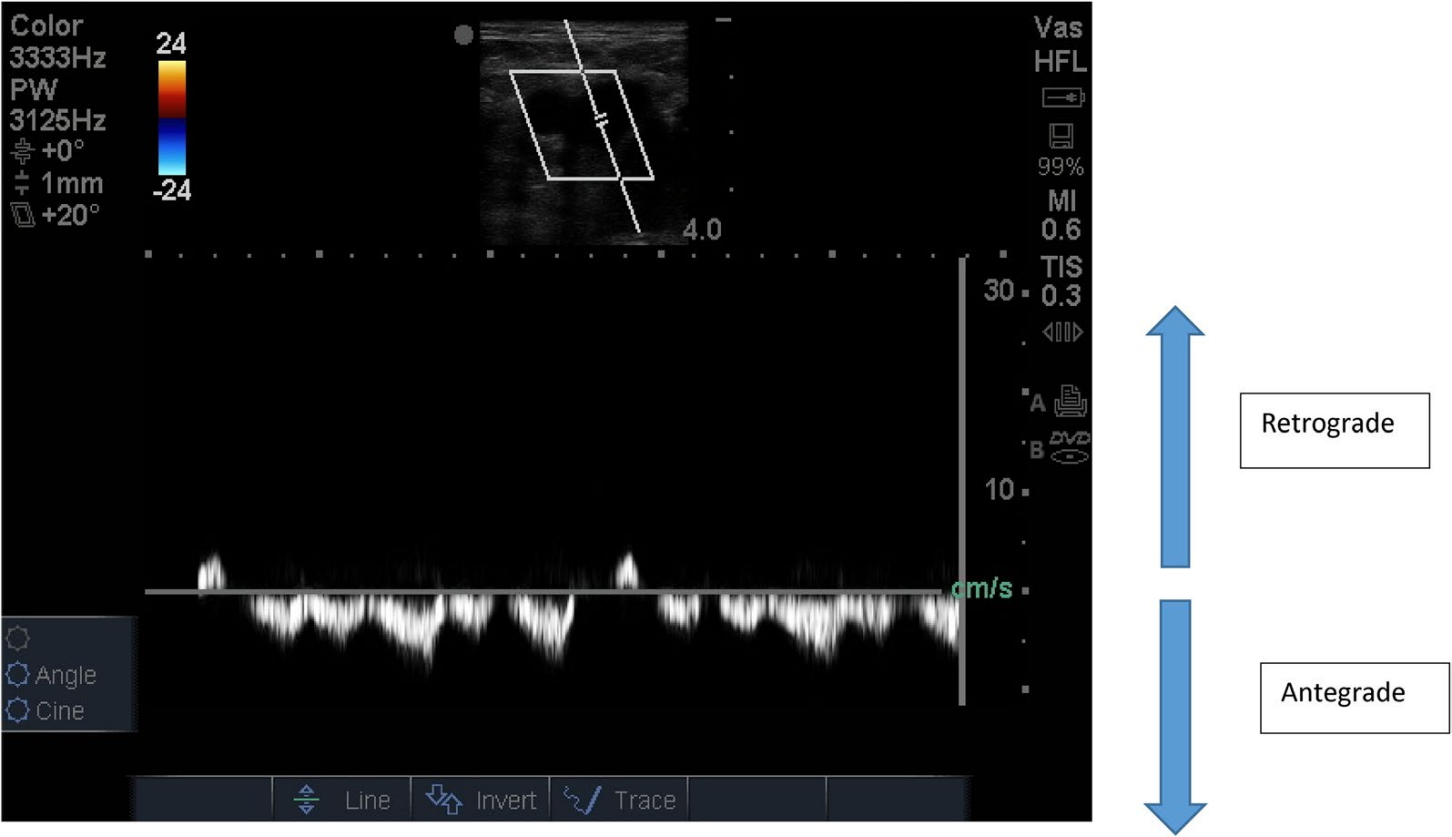
Normal FVD waveform antegrade flow more than retrograde flow with respiratory variation and the flow can be described as antegrade mildly pulsatile uninterrupted pattern

The FVD was obtained with the patient in supine position with a linear array probe. Common femoral vein was identified just 2–3 cm below the inguinal ligament and it was examined in both short and long axis with angle correction within 60°. Normal FVD was defined as antegrade mildly pulsatile uninterrupted pattern with respiratory variation and retrograde flow of less than 1/3rd of antegrade flow (Fig. 1).

**Fig. 2:**
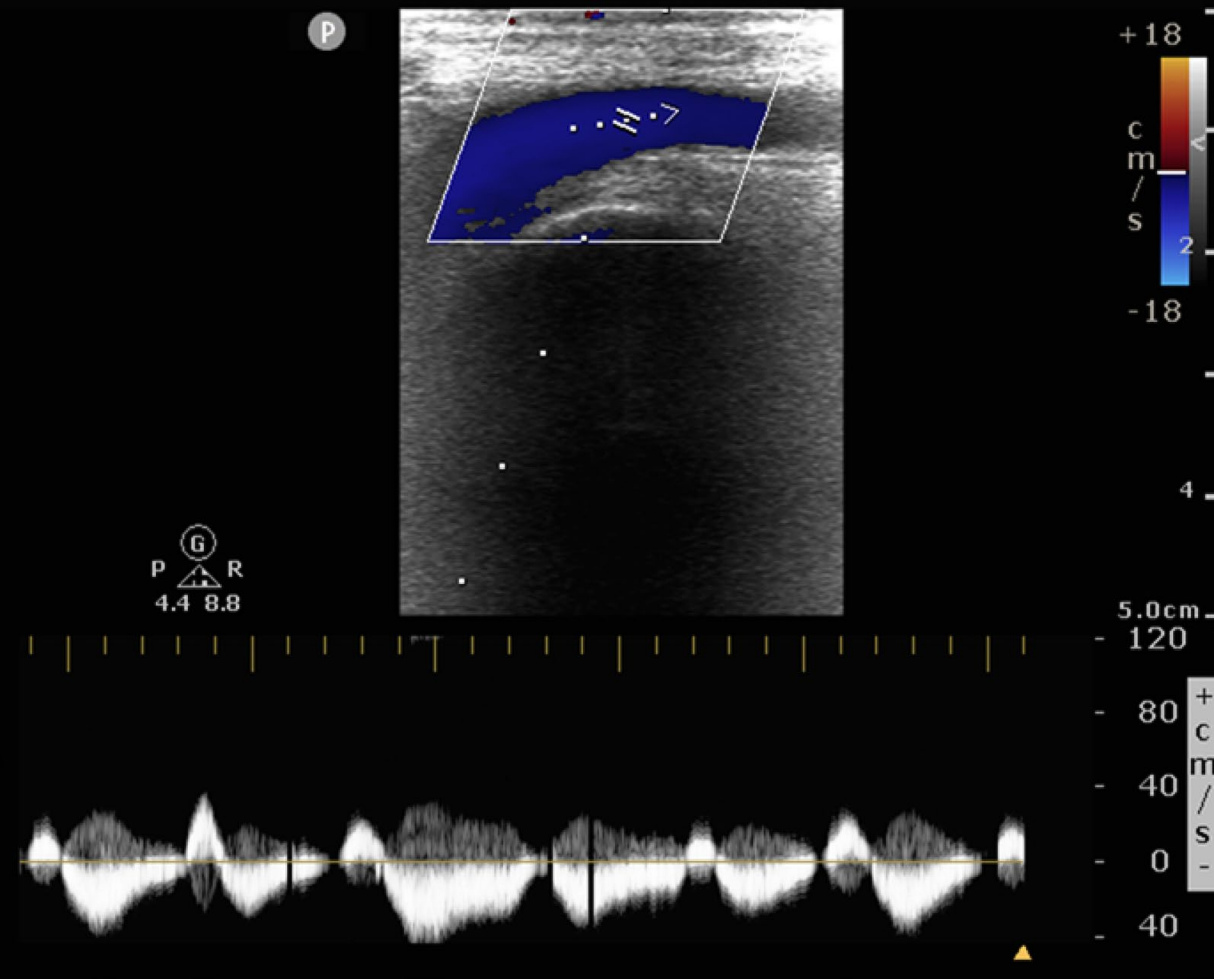
FVD suggestive of venous congestion. Retrograde velocity of more than 10 cm/s (pulsatile with flow reversal)

**Fig. 3:**
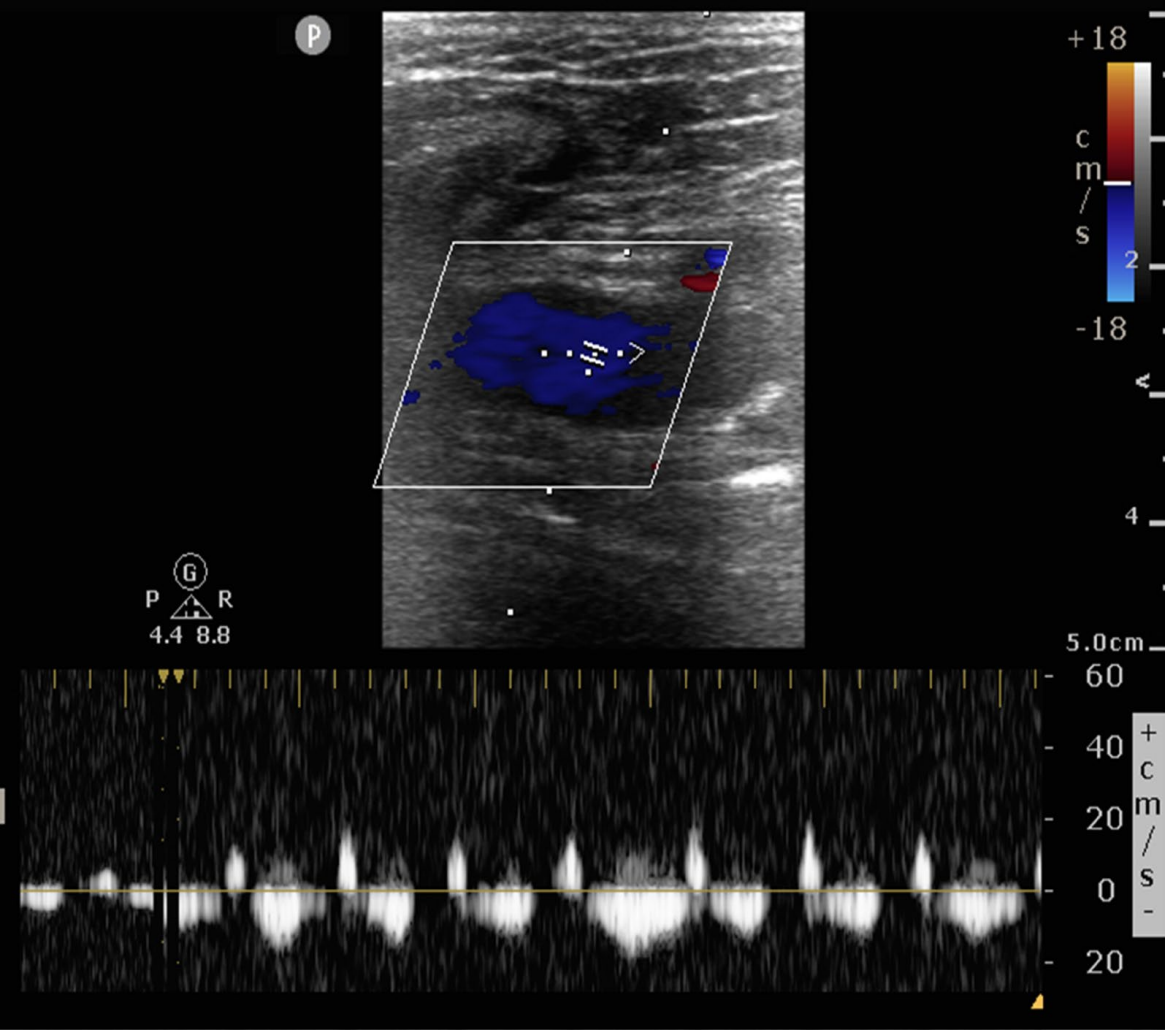
FVD suggestive of venous congestion. Retrograde flow 1/3rd more than antegrade flow (pulsatile with flow reversal)

FVD was considered significant or suggestive of venous congestion if either of the criteria was fulfilled:

1. Pulsatile in nature
2. Retrograde flow velocity of more than 10 cm/s
3. Retrograde flow velocity being more than 1/3rd of antegrade flow velocity (flow reversal) (Figs. 2, 3).

Pulmonary artery pressure was estimated using continuous wave (CW) Doppler of the tricuspid regurgitation (TR) tracing. This method allows measurement of the peak regurgitant velocity which represent the pressure difference between the right ventricle and right atrium. The simplified Bernoulli equation [*P* = 4(TRmax)^2^] was used to calculate this pressure difference using peak TR velocity. This method was used to calculate systolic pulmonary artery pressures. Mean PAP were approximated from the systolic PAP (SPAP) using the following formula: mPAP = 0.61 × SPAP + 2 mmHg [6].

## Study outcomes

The primary outcome was the correlation between FVD and the VExUS score. Based on the pulsatility and congestion in the hepatic vein, portal vein and inferior vena cava, VExUS grading is done from 0 to 3, where grade 0 means no congestion and grade 1–3 means increasing degree of congestion. The FVD was described as normal, pulsatile, or pulsatile with flow reversal. Pulsatile and pulsatile with flow reversal FVD patterns were considered suggestive of venous congestion. A CVP of 12 mmHg or more was considered indicative of venous congestion, a threshold used in much of the literature.

Secondary outcomes were the correlation between FVD or VExUS score with CVP; correlation with other markers of volume overload such as edema and pulmonary artery pressure; effect of mechanical ventilation on correlation between FVD and VEXUS score.

Edema was graded according to the criteria:

- Grade 1: Absence of any edema.
- Grade 2: Mild bilateral ankle edema.
- Grade 3: Moderate ankle and lower leg edema.
- Grade 4: Generalized edema (anasarca) with or without pulmonary edema.

Grade 2–4 was considered edematous condition.

Pulmonary artery pressures were measured by echocardiography.

## Statistical methods

A sample size of 112 patients was required for an expected agreement in diagnosis (kappa value) between FVD and VEXUS of 0.7 with an estimated prevalence of an elevated CVP of 30%. Data were analyzed using SPSS Statistics for Windows, Version 21.0. Baseline patient characteristics were expressed using mean and standard deviation or median with interquartile range for continuous variables, frequency with percentage for categorical variables. The association between categorical variables (like VExUS grade; raised CVP and abnormal femoral venous pulsatility) was analyzed using Chi-square test or Fisher exact test. Agreement in diagnosis was analyzed using Cohen’s Kappa test. Sensitivity, specificity, positive predictive value, negative value and accuracy was calculated along with 95% confidence interval, keeping CVP as a gold standard. A *P* value* < 0.05* was considered statistically significant.

## Results

A total of 107 patients were enrolled for the study. Mean age of the patients was 55.67 ± 12.76 with 78 males (72.9%). The median EURO score was 2 (IQR 1,3) and 56 (52.3%) were mechanically ventilated (Table 1). Among these patients, abnormal FVD were present in 49% of patients.

**Table 1:** Showing baseline patient characteristics

Variable	Values (*n* = 107)
Age	55.67 ± 12.76
Gender (male)	78 (72.9)
Euroscore	2 (1,3)
Type of surgery	
∙CABG	70 (65.4%)
∙Valve repair/replacement	32 (30%)
∙Others	
∙Septal myectomy	5 (4.6%)
∙ASD closure	
∙Bentall procedure	
∙Pulmonary thromboendarterectomy	
Ventilator	56 (52.3)
VExUS grade	
Grade 0	63 (58.9)
Grade 1	15 (14)
Grade 2	14 (13.1)
Grade 3	15 (14)
PA pressure (mmHg)	30.94 ± 5.46
CVP (mmHg)	9.81 ± 6.55
TAPSE (mm)	13.18 ± 2.25

Continuous variables expressed as mean and standard deviation. Categorical variables expressed as frequency and percentage

*CABG* coronary artery bypass graft, *TAPSE* tricuspid annular plane systolic excursion

There was significant correlation between VExUS, CVP and FVD in interpreting venous congestion (Table 2) Over 80.5% of patients with elevated CVP had abnormal pulsation in FVD (*P < 0.001*) and 78% had VExUS grade 1 to grade 3 congestion (*P < 0.001*). Similarly, 86.4% of patients having VExUS grade 1 to grade 3 congestion had abnormal pulsation in FVD (*P < 0.001*). Among patients with a VExUS grade between1 and 3, 86.4% had abnormal FVD Doppler patterns (Table 2). Similarly, 77.8% of patients who had a VExUS grade of 0 had normal femoral venous Doppler waves.

**Table 2:** Comparison of results based on CVP, FVD and VExUS

	CVP elevated	CVP normal	*P* value
FVD abnormal	33 (80.5%)	19 (28.8%)	< 0.001
FVD normal	8 (19.5%)	47 (71.2%)	

	CVP elevated	CVP normal	*P* value
VExUS grade 1–3 (congestion)	32 (78%)	12 (18.2%)	< 0.001
VExUS grade 0 (normal)	9 (22%)	54 (81.8%)	

	VExUS grade 1–3 (congestion)	VExUS grade 0 (normal)	*P* value
FVD abnormal	38 (86.4%)	14 (22.2%)	< 0.001
FVD normal	6 (13.6%)	49 (77.8%)	

Statistical test used: Chi-square test

Agreement in diagnosis of venous congestion based on VExUS grading, central venous pressure and femoral vein Doppler were analyzed separately for ventilated, non-ventilated and overall patients (Table 3) There was moderate agreement between FVD with VEXUS, FVD with CVP and VEXUS with CVP both in ventilated (Kappa value of 0.58, 0.58 and 0.75, respectively) and non-ventilated patients (Kappa value of 0.64, 038 and 0.41, respectively).

**Table 3:** Agreement between different measures of venous congestion

	Measure of agreement (kappa value)	*P* value
FVD with CVP	0.49	< 0.001
FVD with Vexus	0.62	< 0.001
Vexus score with CVP	0.59	< 0.001
Ventilated patients		
FVD with CVP	0.58	< 0.001
FVD with Vexus	0.58	< 0.001
Vexus score with CVP	0.75	< 0.001
Non-ventilated patients		
FVD with CVP	0.38	0.005
FVD with Vexus	0.64	< 0.001
Vexus score with CVP	0.41	0.003

**Fig. 4:**
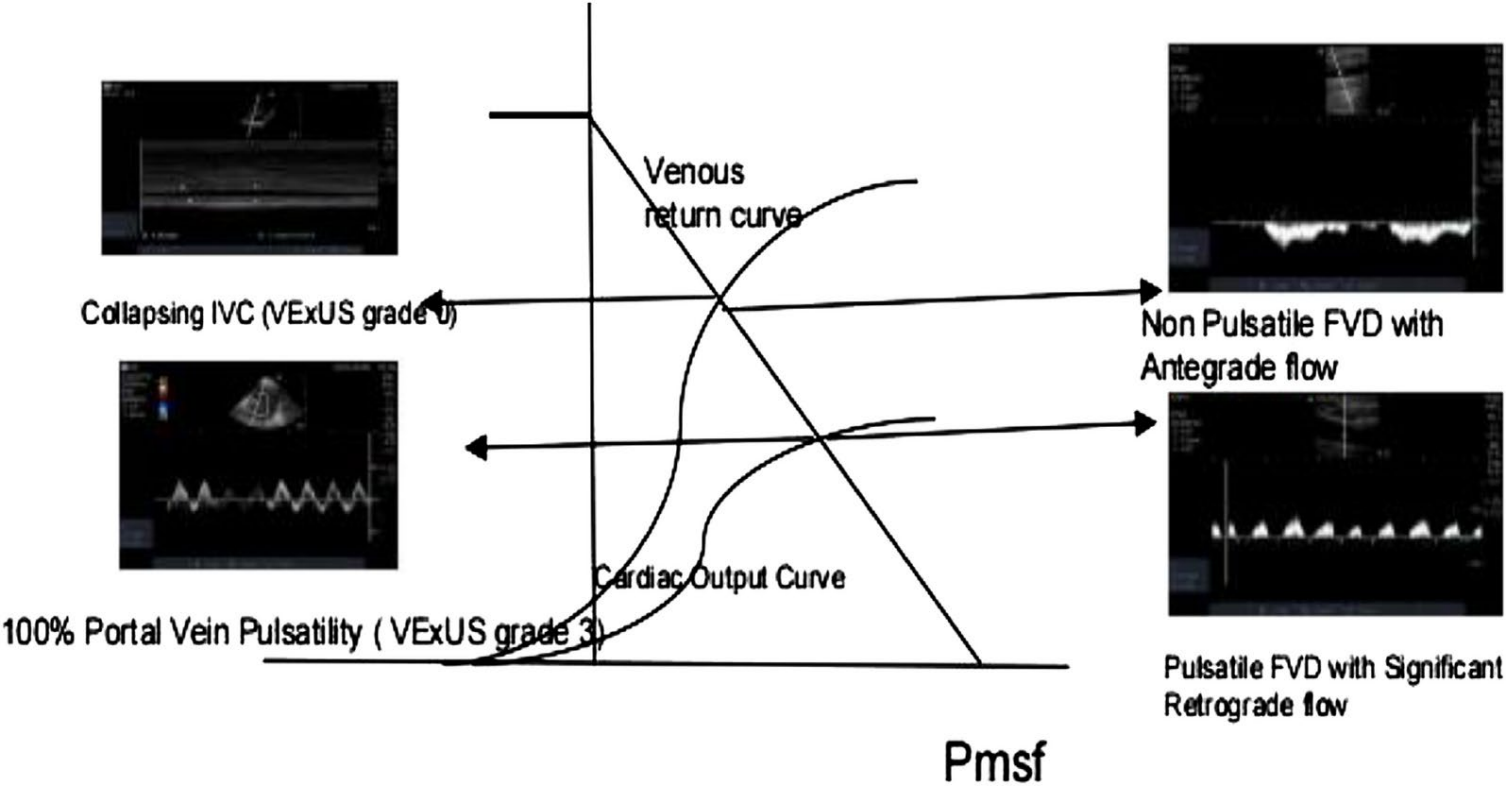
Proposed Guytonian curve on correlation of VExUS with FVD. This is a modified Guytonian curve as a hypothesis to our findings. The normal Frank starling curve with venous return curve intersecting at ascending portion shows a collapsing IVC (vexus grade 1) correlating with antegrade predominantly monophasic Femoral venous Doppler flow and the lower Frank Starling curve with venous return intersection at flat portion shows pulsatile portal vein correlating with VExUS grade 3 and corresponding pulsatile femoral venous Doppler without respiratory variation.

The diagnostic accuracy of VExUS and FVD for predicting congestion was analyzed keeping CVP as a gold standard (Table 4). VExUS had a sensitivity of 78% (95% CI of 62.4 to 89.4) and specificity of 81.8% (95% CI of 70.39 to 90.24), FVD had a sensitivity of 80.5% (95% CI of 65.1 to 91.1) and specificity of 71.2% (95% CI of 58.7 to 81.7). Similar findings were also seen in ventilated and non-ventilated patients (Fig. 4).

**Table 4:** Diagnostic accuracy of FVD and VExUS

Overall (*n* = 107)	Sensitivity % (95% CI)	Specificity% (95% CI)	PPV (95% CI)	NPV (95% CI)	Accuracy % (95% CI)
FVD	80.5 (65.1–91.1)	71.2 (58.7–81.7)	63.46 (53.5–72.3)	85.45 (75.6–91.8)	74.7 (65.4–82.6)
VExUS	78 (62.4–89.4)	81.8 (70.39–90.24)	72.7 (60.9–82)	85.7 (76.9–91.5)	80.37 (71.5–87.4)
Ventilated patients (*n* = 56)					
FVD	83.3 (58.5– 96.4)	78.9 (62.6– 90.4)	65.2 (49.4– 78.2)	90.9 (77.8– 96.6)	80.36 (67.5–89.7)
VExUS	83.3 (58.5– 96.4)	92.1 (78.6– 98.3)	83.3 (62.3– 93.7)	92.1 (80.5– 97)	89.2 (78.1– 95.9)
Non-ventilated patients (*n* = 51)					
FVD	78.2 (56.3– 92.5)	60.7 (40.5– 78.5)	62 (49.6– 73.1)	77.2 (59.7– 88.6)	68.6 (54.1– 80.8)
VExUS	73.9 (51.5– 89.7)	67.8 (47.6– 84.1)	65.3 (51.1– 77.3)	76 (60.3– 86.8)	70.5 ( 56.1– 82.5)

When analyzed for correlation between peripheral edema and FVD (Table 5), 32.7% of patients with abnormal femoral vein doppler had edema and only 1.8% of patients with normal FVD had edema (*P < 0.001*). However, 50% of patients with raised PA pressure had abnormal FVD showing no significant correlation between FVD and PA pressure (*P* value 0.37).

**Table 5:** Correlation of FVD with peripheral signs of congestion and PA pressure

	FVD abnormal	FVD normal	*P* value
Edema	17 (32.7%)	1 (1.8%)	< 0.001
No edema	35 (67.3%)	54 (98.2%)	

	PA pressure raised	PA pressure normal	*P* value
FVD abnormal	49 (50%)	3 (37.5%)	0.37
FVD normal	49 (50%)	5 (62.5%)	

Statistical test used: Fisher exact test

## Discussion

**Fig. 5:**
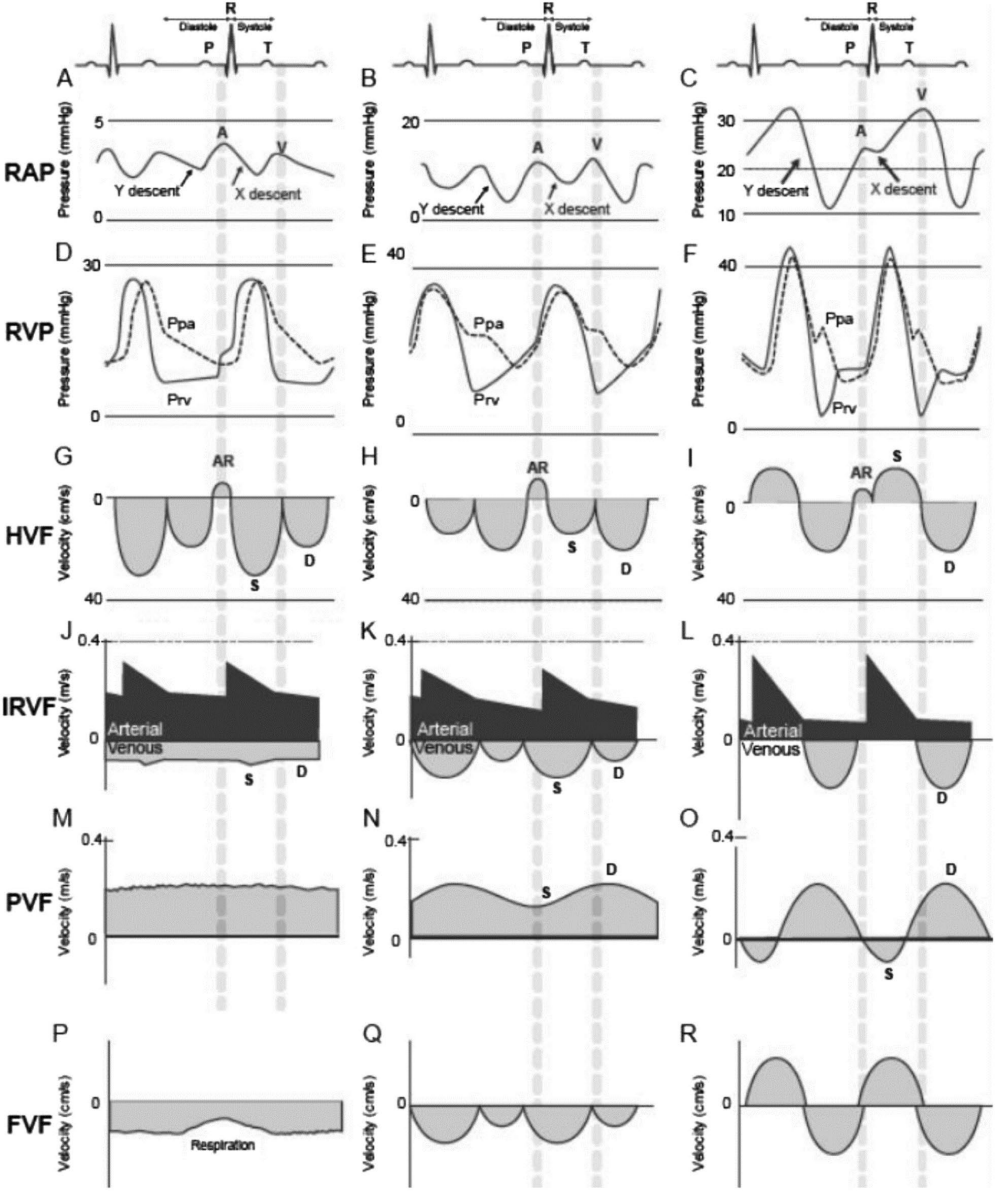
Correlation between right atrial pressure waveform (RAP), right ventricular pressure waveform (RVP), hepatic venous flow (HVF), interlobar renal venous flow (IRVF), portal venous flow (PVF) and femoral venous flow (FVF) with progressive right ventricular (RV) dysfunction and venous congestion in normal patients (**A**, **D**, **G**, **J**, **M**, **P**). Typical patterns are commonly observed in patients with mild (**B**, **E**, **H**, **K**, **N**, **Q**) and severe (**C**, **F**, **I**, **L**, **O**, **R**) RV dysfunction. *AR* atrial reversal Doppler flow velocity; *D* diastolic Doppler flow velocity; *Ppa* pulmonary artery pressure, *Prv* right ventricular pressure, *S* systolic Doppler flow velocity. (Adapted with permission of Couture et al. [9])

Our results show a strong correlation between femoral venous Doppler and splanchnic solid organ venous Doppler (VExUS). This comes as no surprise as the femoral veins are in a direct axis to the IVC and the right atrium, hence significant flow perturbations should be noted in parallel. As right atrial pressures elevate causing venous congestion, the dampening of central pulsatility attenuates, converting normal continuous venous flow to interrupted pulsatile venous flow, essentially the CVP tracing becomes reflected in the peripheral venous system (Fig. 5). This phenomenon was observed in 1925 by Kerr et al. [7] who observed the occurrence of transmitted peripheral venous pulsation in congestive cardiac failure patients. The femoral vein—particularly the right femoral vein—is an extension of IVC with a relatively straight course and reflects a window to estimate IVC/right atrial dynamics. The size and continuum relationship explains the Doppler profile changes corresponding to the level of congestion (Table 6).

**Table 6:** Correlation of FVD with CVP and VExUS score (ventilated patients)

	CVP elevated	CVP normal	*P* value
FVD abnormal	15 (83.3%)	8 (21.1%)	< 0.001
FVD normal	3 (16.7%)	30 (78.9%)	

	CVP elevated	CVP normal	*P* value
VExUS grade1-3 (congestion)	15 (83.3%)	3 (7.9%)	< 0.001
VExUS grade 0 (normal)	3 (16.7%)	35 (92.1%)	

	VExUS grade 1–3 (congestion)	VExUS grade 0 (normal)	*P* value
FVD abnormal	15 (83.3%)	8 (21.1%)	< 0.001
FVD normal	3 (16.7%)	30 (78.9%)	

Statistical test used: Fisher exact test

**Fig. 6:**
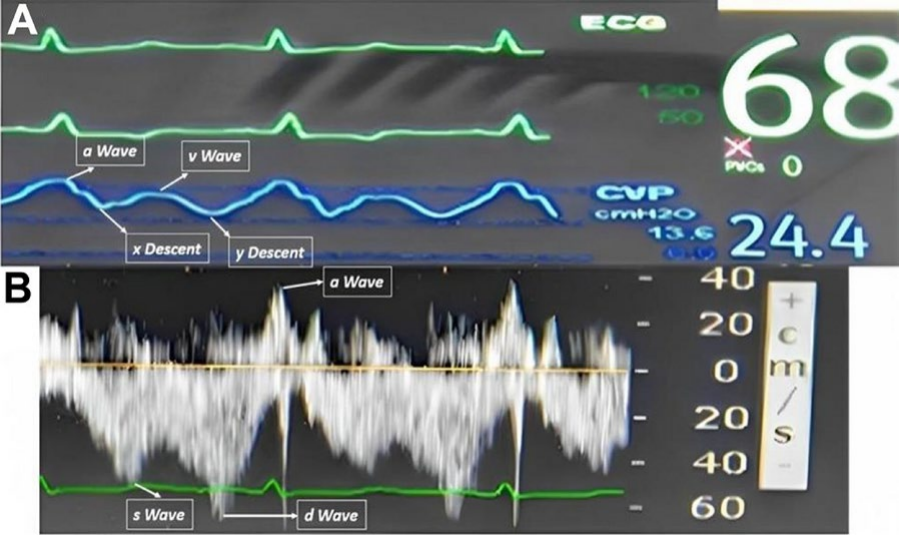
FVD and CVP waveform in same patient for comparison. **A** High CVP (mean = 24.4 cmH_2_O) with ECG correlation. **B** Corresponding femoral venous Doppler with ECG correlation. As the central pressure rises, the vein rounds out; giving femoral venous Doppler a pulsatile pattern. FVD is an inverse representation of CVP waveform. As the CVP rises and stretches the atrium, the pattern of CVP changes and FVD reflects the same. When CVP rises, Y descent exceeds X descent; accordingly, the FVD ‘d Wave’ being more prominent than the ‘s Wave’. FVD shows a prominent retrograde ‘a Wave’ with velocity of 20 cm/s indicating venous congestion

When there is no significant venous congestion, peripheral veins tend to be smaller, non-plethoric, owing to low intraluminal pressure. The Doppler profile demonstrates an indistinct pattern with respiratory variation. As the intraluminal pressure rises due to venous congestion, the vein rounds out and the Doppler profile exhibits a pulsatile pattern with less respiratory variation and becomes a representation of the CVP waveform. With severe congestion and impaired right ventricular systolic function and tricuspid regurgitation, the CVP waveform shows prominent V and Y waves, giving it a bidirectional pulsatile pattern on FVD (Fig. 6). This leads to a pulsatile FVD, corroborating higher grades of VExUS (grade 1–3) and CVPs above 12 mmHg. In healthy individuals retrograde flow is observed, but we set a criteria of 10 cm/s of retrograde flow velocity as an indicator of congestion based on case control study by McClure et al. who observed that healthy patients had a peak retrograde velocity of 4.7 to 8.4 cm/s (mean 6.4 cm/s) versus 12.9 to 50.4 cm/s (mean 31.2 cm/s) in patients with cardiac dysfunction in lower limb venous Doppler [8]. The presence of femoral vein blood velocity pulsatility is an indicator of elevated right atrial pressure which is transmitted in the periphery which include the hepatic vein, the intrarenal vein, the portal and splenic vein, the femoral vein and even the popliteal vein [9]. Our study supports a correlation between FVD and IVC in both ventilated and non-ventilated patient. In view of confounders in a ventilated patient causing unreliability of IVC (increased intraabdominal pressure causing collapse of IVC); we presume that same set of drawbacks exist with FVD as well. Studies on FVD have been performed and correlated with right atrial pressure in both ventilated [10] and non-ventilated patients [11]. Peripheral venous Doppler signal are very simple and easy to obtain and provide to the clinician a very important information regarding right heart function [5].

**Fig. 7:**
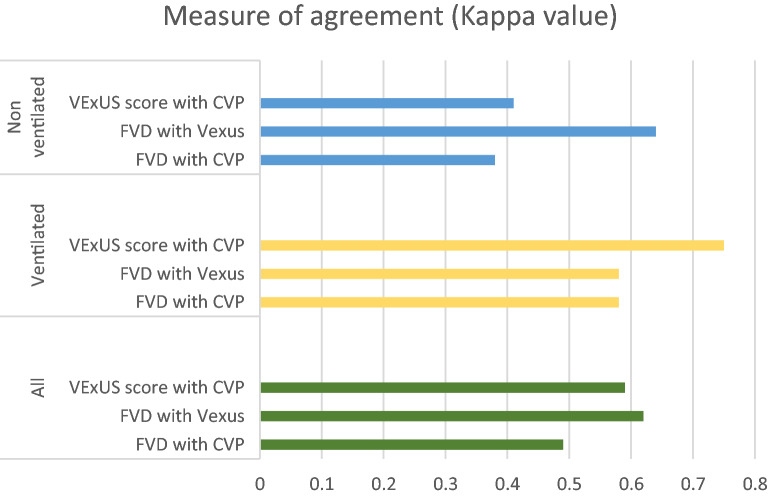
Measure of agreement between VExUS, FVD and CVP

Ours is not the first group to investigate the association between venous congestion and femoral Doppler. Abu-Yousef et al. found a strong correlation between elevated right atrial pressure with good specificity, but lacked sensitivity [11]. While Cozcoluella et al. did not find a strong correlation between CVP and FVD, they did find a significant correlation with the presence of a V wave [12]. To our knowledge this is the first study to investigate and correlate the relationship of FVD with the VExUS score (Fig. 4). As femoral vein has anatomical and physiological continuum with IVC, it might reflect the changes in IVC thereby correlating with VExUS score better then CVP hence our study has shown significant association with VExUS score over CVP (Fig. 7).

## Conclusion

This study adds one more entry to the study of venous congestion markers as we do not have a single gold standard entity. The potential advantage of CFV is that it might not be subjected to the controversy surrounding IVC visualization (short Axis vs long Axis) [13, 14], along with easier anatomical accessibility and a shorter learning curve (Table 7).

**Table 7:** Correlation of FV with CVP and Vexus score (non-ventilated patients)

	CVP elevated	CVP normal	*P* value
FVD abnormal	18 (78.3%)	11 (39.3%)	0.005
FVD normal	5 (21.7%)	17 (60.7%)	

	CVP elevated	CVP normal	*P* value
VExUS grade1-3 (congestion)	17 (73.9%)	9 (32.1%)	0.003
VExUS grade 0 (normal)	6 (26.1%)	19 (67.9%)	

	VExUS grade 1–3 (congestion)	VExUS grade 0 (normal)	*P* value
FVD abnormal	23 (88.5%)	6 (24%)	< 0.001
FVD normal	3 (11.5%)	19 (76%)	

Statistical test used: Chi-square test/Fisher exact test

### Limitations

There are several pitfalls and limitations of femoral venous Doppler. Those include:

- FVD measurement is advocated in supine position hence might not be feasible in orthopneic patient
- FVD mirrors IVC hence the fallacies of IVC as a marker of venous congestion specifically in higher intraabdominal pressure, cirrhosis and respiratory distress make it unreliable
- Deep venous thrombosis
- Varicose veins with saphenofemoral junction incompetence might give confounding results
- Femoral venous pulsations can be seen in healthy individuals but typically without systolic reversal. We have used CVP as the gold standard which has its own limitations in the clinical settings.

In our study, we have taken CVP as gold standard marker for venous congestion which has it’s set of limitations.

## Supplementary Information

Additional file 1. VExUS methodology

## Data Availability

Baseline demographic details were obtained from medical records. Deidentified USG examination images for VExUS score, femoral vein pulsatility has been preserved in digital library.

## Abbreviations

FVD: Femoral venous Doppler
VExUS: Venous Excess Ultrasound Score
IVC: Inferior vena cava
CVP: Central venous pressure
PA: Pressure pulmonary artery pressure
PPV: Positive predictive value
NPV: Negative predictive value
