# Supplementary material for: Femoral vein pulsatility: a simple tool for venous congestion assessment
Source: Ultrasound J. 2023 May 10;15:24. doi: 10.1186/s13089-023-00321-w (PMC10172460; doi:10.1186/s13089-023-00321-w)

**Annexure 1- VExUS methodology**

The IVC is interrogated in long axis along the intrahepatic segment and a visual average was done. Respiratory variation was defined as a 20% or more change in diameter in the long axis.

Grade 0: <5 mm with respiratory variation Grade I: 5–9 mm with respiratory variation Grade II: 10–19 mm with respiratory variation Grade III: >20 mm with respiratory variation

|  |  |
| --- | --- |
|  | 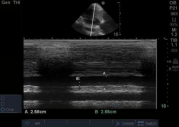 |

Grade IV: >20 mm with minimal or no respiratory variation

Grade III IVC

Hepatic vein (HV): interrogation by pulsed wave Doppler, identification and analysis of A, S, and D waves:

Grade 0: normal S > D

Grade I: S < D with antegrade S

Grade III: S flat or inverted or biphasic trace

We have chosen the approach that the S wave is normally the larger of the two negative deflections.


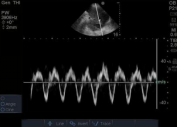


Hepatic Venous doppler with biphasic waveform; implying severe congestion. Portal vein Doppler (PD)

Portal vein (PV) interrogation Grade 0: <0.3 pulsatility index Grade I: 0.3–0.49 pulsatility index Grade III: 0.5–1.0 pulsatility index

Pulsatility index is calculated as (Vmax − Vmin)/Vmax


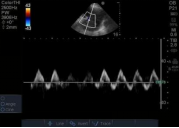


Pulsatile Portal vein with 100% pulsatility, signifying grade3 congestion

The individual Doppler staging has been compiled into VEXUS staging of venous congestion.

Venous Excess Ultrasound Score (VEXUS) Grade 0: IVC grade <III, HD grade 0, PV grade 0 Grade I: IVC grade IV, but normal HV/PV pattern

Grade II: IVC grade IV with mild flow pattern abnormalities in HV/PV

Grade III: IVC grade IV with severe flow pattern abnormalities in HV/PV

Relationship between the CVP and the venous Doppler signals are more correlated with the CVP waveform than the values as illustrated by Tang et al (Tang WH, Kitai T. Intrarenal Venous Flow: A Window Into the Congestive Kidney Failure Phenotype of Heart Failure? JACC Heart Fail 2016; 4: 683-6. Doi: 10.1016/j.jchf.2016.05.009)


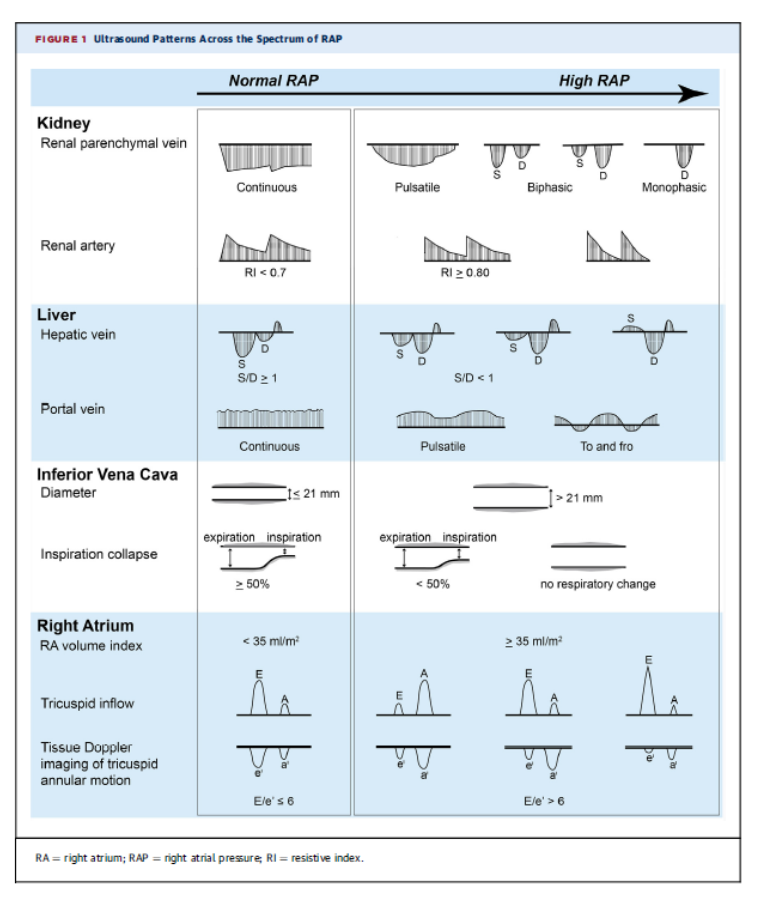

Supplement: Supplementary file 1 — Additional file 1. VExUS methodology [file 13089_2023_321_MOESM1_ESM.docx]
